# Supplementary material for: Systematically attenuating DNA targeting enables CRISPR-driven editing in bacteria
Source: Nat Commun. 2023 Feb 8;14:680. doi: 10.1038/s41467-023-36283-9 (PMC9908933; doi:10.1038/s41467-023-36283-9)
Supplement: Supplementary file 1 — Supplementary Information [file 41467_2023_36283_MOESM1_ESM.pdf]

SUPPLEMENTARY INFORMATION

Systematically attenuating DNA targeting enables CRISPR-driven editing in bacteria

Daphne Collias, Elena Vialetto, Jiaqi Yu, Khoa Co, Éva d.H. Almási, Ann-Sophie Rüttiger, Tatjana Achmedov, Till Strowig, Chase L. Beisel

Correspondence: chase.beisel@helmholtz-hiri.de (to C.L.B.)

Supplementary figures and legends

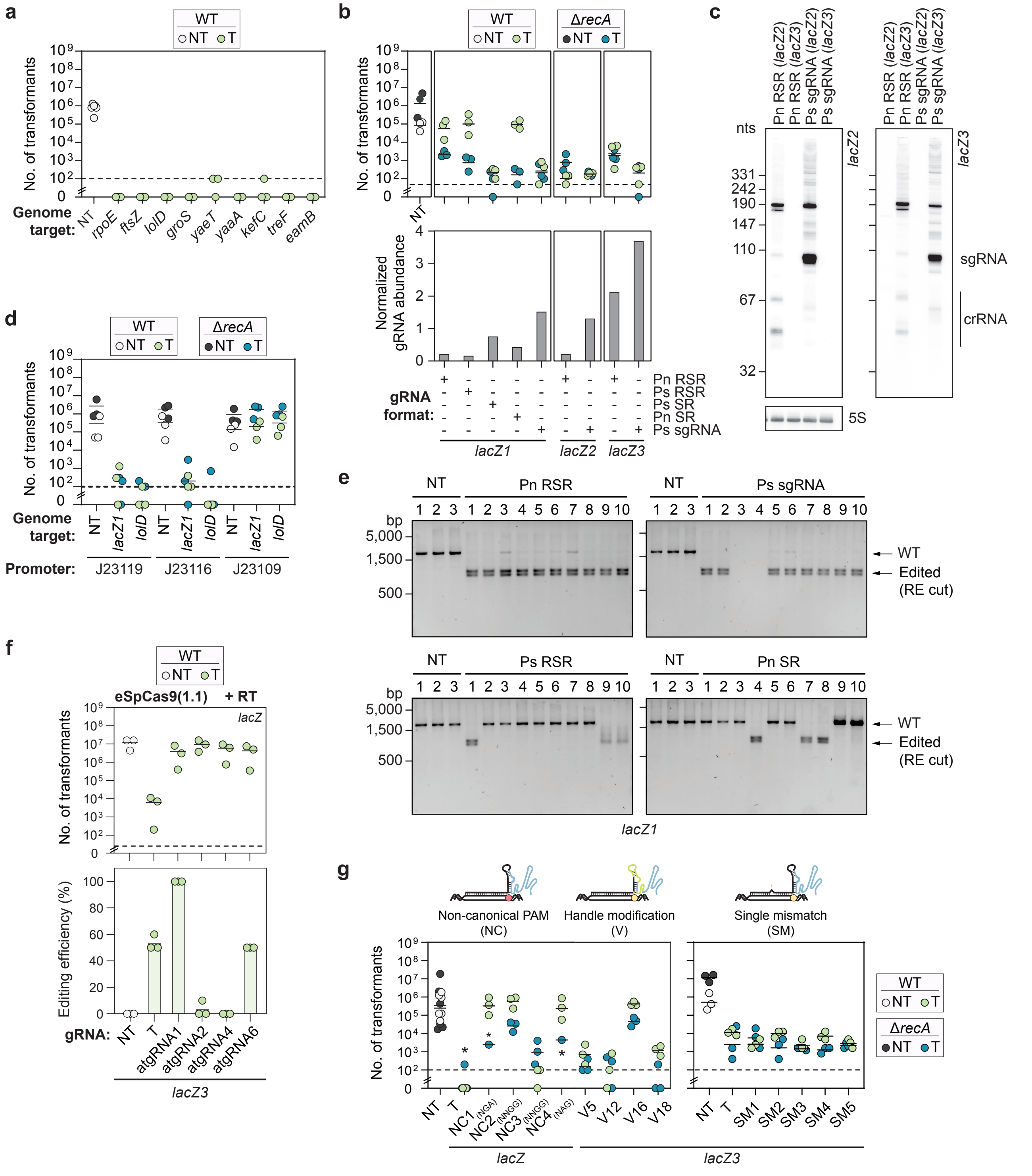

Supplementary Figure 1. Assessing genome targeting and editing activity in *E. coli* with Cas9. a) Transformation assay in the WT strain of *E. coli* with Cas9 and sgRNAs. **b)** Transformation assays in the WT and Δ*recA* strain of *E. coli* with Cas9 and *lacZ* gRNA formats used for northern blotting analysis. **c)** Northern blotting analysis with the *lacZ2* and *lacZ3* probe. 5S was used as a control on the same gel and shown below the *lacZ2* and *lacZ3* probed gels. The middle lane was run with unrelated RNA extracted from *E. coli*. **d)** Transformation assay in the WT and Δ*recA* strain of *E. coli* with Cas9 with genome targeting sgRNAs expressed from different constitutive synthetic promoters: J23119, J23116, and J23109. **e)** Gel readout of restriction enzyme digestion reactions on transformants with gRNAs and RTs from Fig. 1d. The WT and unedited amplicons are expected to be 1.9kb whereas if editing occurred and the RE recognition site was installed then two digestion fragments of ~1.1 kb and ~900 bp. **f)** Genome editing assay in *E. coli* using selected atgRNAs from Fig. 1d using eSpCas9(1.1). Individual dots for the editing efficiencies are based on screening 3 colonies for NT samples or 10 colonies for targeting samples from one biological replicate. Bars indicate the mean of the dots. The targeting (T) condition for one biological replicate yielded only two colonies that could be screened. **g)** Transformation assay in the WT and Δ*recA* strain of *E. coli* with Cas9 with modified gRNAs. * indicates that the transformants resulted in a lawn or uncountable colonies. The dashed line in a, b, e, g, and h indicate the limit-of-detection. See Fig. 1 for details. The mean number of transformants (indicated by a horizontal line) was not calculated for samples with biological replicates that fell below the limit of detection. WT indicates wild-type *E. coli* MG1655, Δ*recA* indicates *E. coli* MG1655 Δ*recA*, NT indicates non-targeting, T indicates targeting, and RT indicates repair template.

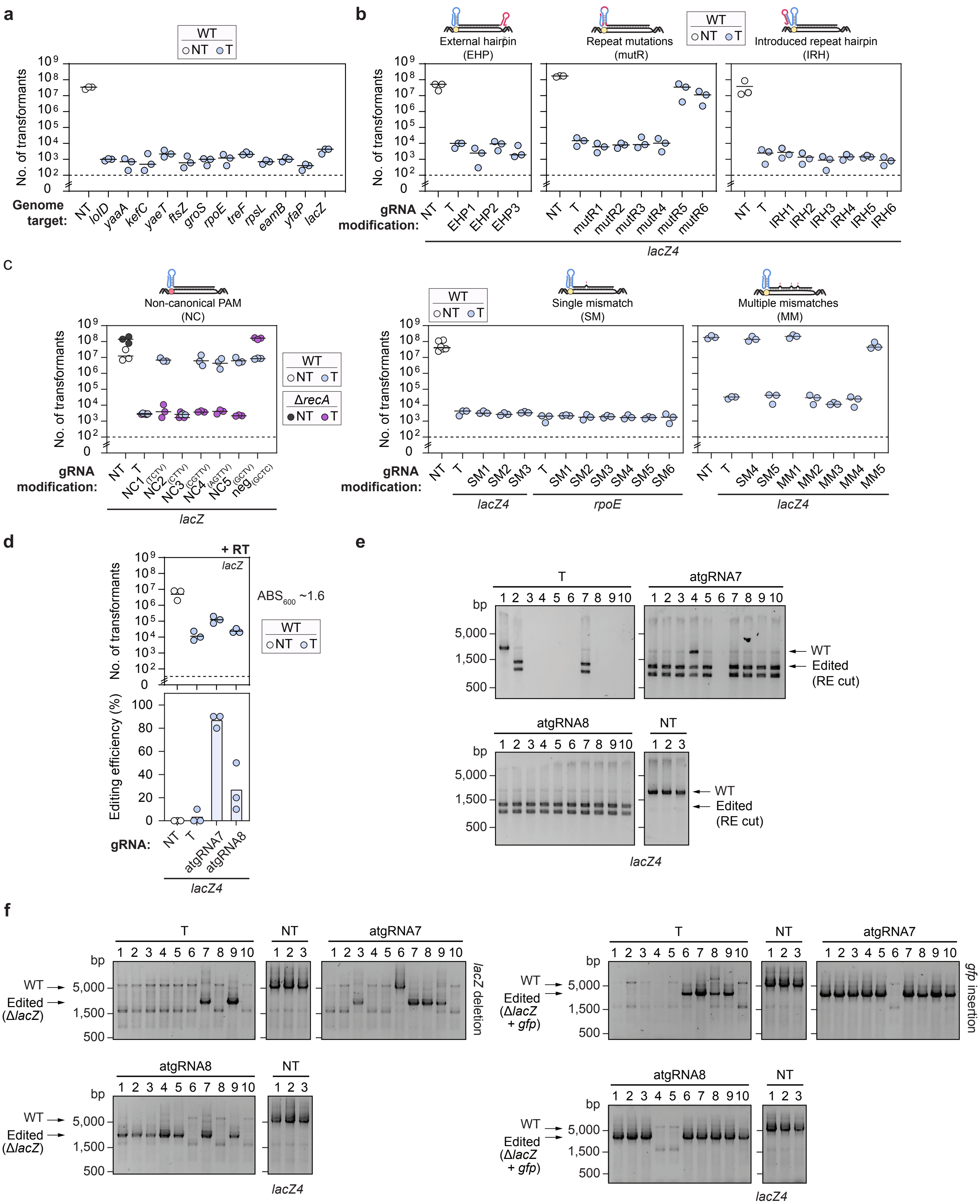
a
WT
NT
T
No. of transformants
Genome target:
NT
lolD
yaaA
kefC
yaeT
ftsZ
groS
rpoE
treF
rpsL
eamB
yfaP
lacZ
b
External hairpin (EHP)
Repeat mutations (mutR)
Introduced repeat hairpin (IRH)
gRNA modification:
EHP1
EHP2
EHP3
mutR1
mutR2
mutR3
mutR4
mutR5
mutR6
IRH1
IRH2
IRH3
IRH4
IRH5
IRH6
lacZ4
c
Non-canonical PAM (NC)
ΔrecA
NC1 (TCTV)
NC2 (CTTV)
NC3 (CGTTV)
NC4 (AGTTV)
NC5 (GCTV)
neg (GCTC)
lacZ
Single mismatch (SM)
Multiple mismatches (MM)
SM1
SM2
SM3
SM4
SM5
SM6
MM1
MM2
MM3
MM4
MM5
rpoE
d
+ RT
ABS600 ~1.6
Editing efficiency (%)
gRNA:
atgRNA7
atgRNA8
e
bp
5,000
1,500
500
WT
Edited (RE cut)
f
Edited (ΔlacZ)
lacZ deletion
Edited (ΔlacZ + gfp)
gfp insertion

Supplementary Figure 2. Assessing genome targeting activity in *E. coli* with Cas12a. a) Targeting in *E. coli* with AsCas12a. **b)** Transformation assay in WT strain of *E. coli* with AsCas12a using atgRNAs with an external hairpin, introduced repeat hairpin, repeat mutations, and single and multiple mismatches in the *lacZ* guide. Single mismatches were also generated for a guide targeting the *rpoE* gene. **c)** Transformation assay in WT and Δ*recA* strain of *E. coli* with AsCas12a and less-preferred PAMs. neg represents a target site adjacent to a non-PAM sequence. **d)** Editing assay to introduce an AclI restriction enzyme site into *lacZ* at ABS_{600} ≈ 1.4 - 1.6. The experiment showed that for AsCas12a editing cells in the stationary phase led to less transformants for the atgRNAs and lower editing efficiency compared to the experiment performed in exponential phase in Fig 3c. Each dot in the lower graph represents the fraction of edited cells for 10 and 3 screened colonies for the targeting and NT samples respectively. Bars indicate the mean of the dots. **e)** Representative gel for screening the insertion of an AclI restriction site in the *lacZ* gene. Related to Fig. 3c. The WT and unedited amplicons are expected to be ~2 kb whereas if editing occurred and the RE recognition site was installed then two digestion fragments of ~1150bp and ~860bp are expected. **f)** Gel readouts for *lacZ* deletion and *gfp* insertion with 750-bp homology arms. For the *lacZ* deletion experiment, the WT and unedited amplicons are expected to be ~5.4 kb whereas if editing occurred and *lacZ* was deleted then a ~2.3 kb fragment is expected. For the *gfp* insertion experiment, the WT and unedited amplicons are expected to be ~5.4 kb whereas if editing occurred and *lacZ* was replaced with *gfp* then a ~3kb fragment is expected. The dashed line in a-d, f indicates the limit-of-detection. See Fig. 1 for details. The mean number of transformants (indicated by a horizontal line) was not calculated for samples with biological replicates that fell below the limit of detection. WT indicates wild-type *E. coli* MG1655, Δ*recA* indicates *E. coli* MG1655 Δ*recA*, NT indicates non-targeting, T indicates targeting, and RT indicates repair template.

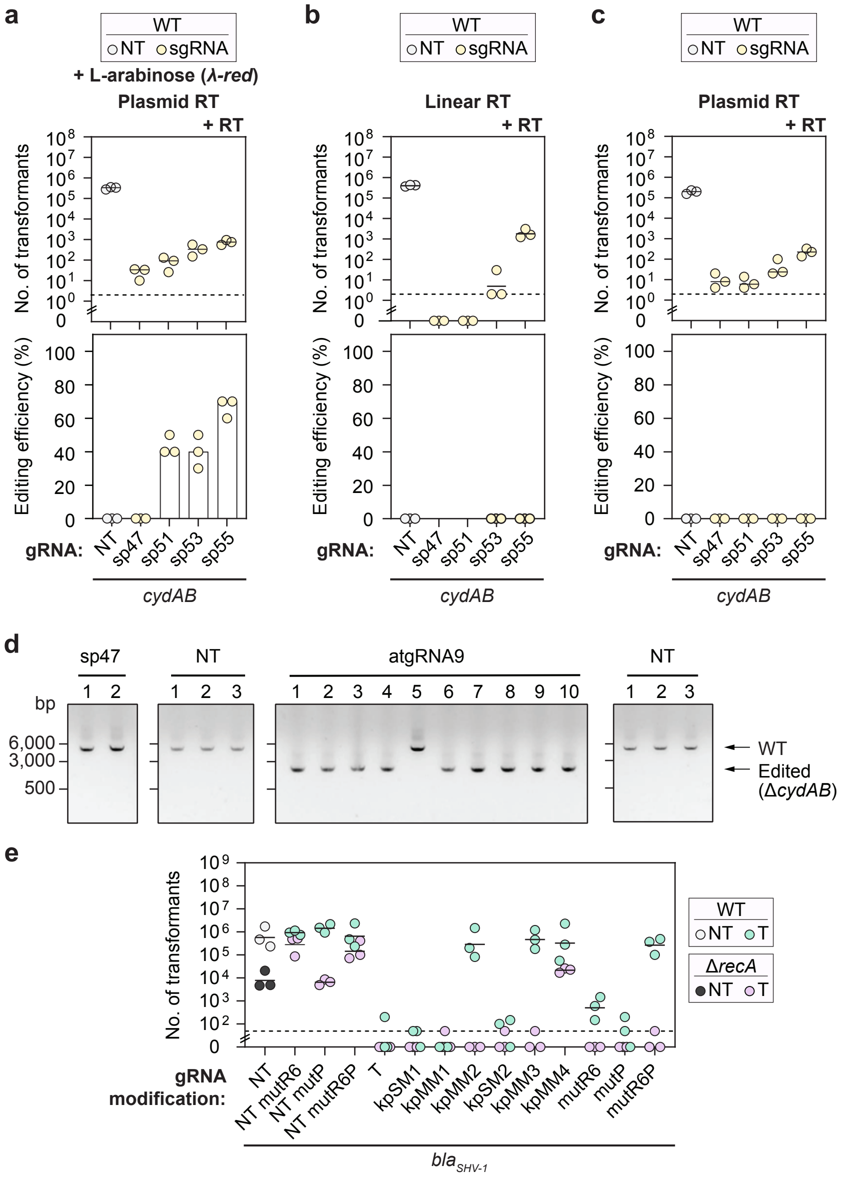

Supplementary Figure 3. Assessing genome targeting and editing activity in strains of *Klebsiella*. a) Editing assay in the WT *K. oxytoca* with a plasmid-based RT and λ-*red* induction. **b)** Editing assay in the WT *K. oxytoca* with linear RT and no λ-*red* induction. **c)** Editing assay in the WT *K. oxytoca* with plasmid-based RT and no λ-*red* induction. **d)** Gel image of colony PCR to screen for *cydAB* deletion from Fig. 4b. The WT and unedited amplicons are expected to be ~4.8 kb whereas if editing occurred and *cydAB* was deleted then a ~2.1 kb is expected. **e)** Targeting assay in *K. pneumoniae* with different guides without the RT. mutP indicates a point mutation in the constitutive promoter, while mutR6P indicates both the mutation of the repeat mutR6 and the promoter mutation mutP. The dashed line in a-c, e indicates the limit-of-detection. See Fig. 1 for

details. The mean number of transformants (indicated by a horizontal line) was not calculated for samples with biological replicates that fell below the limit of detection. Each dot in c represents the editing efficiency determined in d. Bars indicate the mean of the dots. For a, b, and c, WT indicates wild-type *K. oxytoca* MK01. For d, WT indicates wild-type *K. pneumoniae* ATCC 10031. NT indicates non-targeting, and RT indicates repair template.
