# Supplementary material for: Systematically attenuating DNA targeting enables CRISPR-driven editing in bacteria
Source: Nat Commun. 2023 Feb 8;14:680. doi: 10.1038/s41467-023-36283-9 (PMC9908933; doi:10.1038/s41467-023-36283-9)
Supplement: Supplementary file 4 — Description of Additional Supplementary Files [file 41467_2023_36283_MOESM4_ESM.pdf]

Title: Supplementary Data 1.

Description: Strains, plasmids, and oligos used in this work.
